# Surface EMG Sensing and Granular Gesture Recognition for Rehabilitative Pouring Tasks: A Case Study

**DOI:** 10.3390/biomimetics10040229

**Published:** 2025-04-07

**Authors:** Congyi Zhang, Dalin Zhou, Yinfeng Fang, Naoyuki Kubota, Zhaojie Ju

**Affiliations:** 1 School of Computing, University of Portsmouth, Portsmouth PO1 2UP, UK; congyi.zhang@port.ac.uk (C.Z.); dalin.zhou@port.ac.uk (D.Z.); 2 School of Communication Engineering, Hangzhou Dianzi University, Hangzhou 310005, China; yinfeng.fang@hdu.edu.cn; 3 Graduate School of Systems Design, Tokyo Metropolitan University, Tokyo 192-0397, Japan; kubota@tmu.ac.jp

**Keywords:** surface electromyography, gesture recognition, limb motor function, rehabilitation engineering, granular computing

## Abstract

Surface electromyography (sEMG) non-invasively captures the electrical activity generated by muscle contractions, offering valuable insights into motion intentions. While sEMG has been widely applied to general gesture recognition in rehabilitation, there has been limited exploration of specific, intricate daily tasks, such as the pouring action. Pouring is a common yet complex movement requiring precise muscle coordination and control, making it an ideal focus for rehabilitation studies. This research proposes a granular computing-based deep learning approach utilizing ConvMixer architecture enhanced with feature fusion and granular computing to improve gesture recognition accuracy. Our findings indicate that the addition of hand-crafted features significantly improves model performance; specifically, the ConvMixer model’s accuracy improved from 0.9512 to 0.9929. These results highlight the potential of our approach in rehabilitation technologies and assistive systems for restoring motor functions in daily activities.

## 1. Introduction

Gesture recognition is widely applied in human–computer interaction (HCI), especially in rehabilitative and assistive technologies, where understanding human motion intention is crucial. Accurate motion intention recognition is essential for intuitive control of prosthetics and exoskeletons, particularly in clinical and daily rehabilitation settings [1]. Among non-invasive sensing techniques, electromyography (EMG) is the primary method for detecting upper limb motion intentions due to its simplicity and reliability. Combining EMG sensing and machine learning analysis has become the dominant approach in this research area.

Following the foundational work of Parker et al. [2] on EMG-based control, the field of gesture recognition has evolved significantly, particularly with the integration of machine learning (ML) and deep learning (DL) techniques. These advancements have enabled more sophisticated analysis of EMG signals, allowing for the extraction of complex patterns associated with different muscle activations. The application of ML and DL has not only improved the accuracy of gesture recognition but also enhanced the system’s ability to adapt to individual variations in muscle activity, making it more robust for real-world applications. Furthermore, the development of neural networks, as demonstrated by Hudgins et al., has shown promise in classifying EMG patterns with high accuracy, even in the presence of noise and variability. This progress has paved the way for more intuitive and seamless control of prosthetic devices, bringing us closer to achieving natural and efficient human–machine interaction in rehabilitative and assistive technologies.

With the rapid advancement of deep learning, interest in applying these methods to gesture recognition has grown, often surpassing conventional pattern recognition techniques [3]. These approaches rely on large, well-labeled datasets and dexterous network design. Consequently, research has focused on EMG data acquisition and neural network development, including signal processing and network architecture optimization. The integration of wearable sensing technologies with deep learning has greatly advanced gesture recognition, allowing for intuitive, non-invasive identification of complex human motion intentions, and showing significant potential for assistive device control and manipulation.

Despite these advancements, the practical application of sEMG-based rehabilitation remains limited due to the under exploration of clinical factors [4]. A gap persists between the high recognition accuracy achieved in lab settings and the essential clinical requirements for motor function replacement in daily life [5]. While discrete gesture recognition accuracy in lab environments has reached up to 99%, this does not guarantee smooth transitions between gestures, which are crucial for continuous motion in daily tasks [6]. These smooth transitions, closely tied to the intuitiveness of hand movements, are essential for tasks requiring dexterity [7]. One such task, pouring water, demands precise hand and arm coordination and seamless transitions between motions, making it fundamental to daily life. Mastering the pouring tasks is key to regaining independence for individuals undergoing rehabilitation. Yet, despite its significance, the action of pouring water has received limited attention in EMG-based gesture recognition research, matching the gap in the literature.

This study addresses the gap by examining the subtle nuances of pouring water, including variations in pouring angles, directions, and holding forces. By decoding these subtleties through advanced signal processing and computational intelligence, we aim to enhance the accuracy and reliability of EMG-based gesture recognition for pouring tasks in assistive technologies [5,8].

The key contributions of this paper are as follows: (1) A comprehensive analysis of the pouring water gesture, addressing a critical gap in current EMG-based HCI research; (2) the application of combined strategies, including granular computing and feature fusion, to decode complex muscle activities [4]; and (3) improved accuracy in machine learning-based gesture recognition of nuanced pouring actions [6].

The paper is organized as follows: Section 2 reviews related work on hand gesture sensing and analysis. Section 3 outlines the methodology for EMG signal acquisition, experimental protocols, and gesture recognition design. Section 4 presents experimental results for the recognition of pouring water gestures, highlighting the improvements achieved with the proposed strategies. Finally, Section 5 concludes the research and outlines future directions.

## 2. Related Work

### 2.1. Hand Gestures and Benchmarks

Hand gestures can be broadly categorized into static postures and dynamic movements [9]. McNeill recognizes three main groups: iconic gestures, metaphoric gestures, and beat gestures. A more HCI-focused taxonomy developed by Quek distinguishes between communicative and manipulative gestures. Within communicative gestures are acts, symbols, and deictic gestures. Deictic gestures, particularly pointing, are common in HCI and human–robot interaction (HRI) applications. Emblems, such as finger counting, are gestures with common verbal equivalents. Pantomimes, like gestures for menu navigation, are used in favor of speech. Application-specific gestures include boxing moves for video games and arm movements for exercise. Sign language gestures, both for individual letters and words, have received significant attention in research. Micro-gestures involve minimal movement, such as finger movements, while macro-gestures involve large movements of the whole hand. Some researchers have also explored continuous gestures, such as in-air digit writing and alphabet writing. Google’s Soli project demonstrated micro-gestures like finger sliding for scrolling and finger rotation for a virtual dial tool.

With the intuitiveness and importance of hand gestures known, the recognition of hand gestures is a cornerstone of advancements in HCI and HRI. From diverse gesture classifications to the feasibility of wrist-based sensing and the exploration of multimodal gesture datasets, significant progress has been made in improving accuracy and practicality. Several research groups have made important contributions to myoelectric control and hand movement recognition. Atzori et al. created the Ninapro database, a large public dataset of EMG and hand kinematics data from intact and amputee subjects performing various hand movements [10]. Krasoulis et al. investigated combining EMG with inertial measurements to improve prosthetic hand control, demonstrating that this multimodal approach can reduce the number of sensors needed [11]. Jarque-Bou et al. developed a calibrated kinematic dataset of hand movements and grasps from 77 subjects performing 40 movements [12]. Meehan et al. studied user adaptation in prosthetic finger control, showing that even with intuitive decoders, users can improve performance with practice [13]. The hand gestures and movements included in these datasets were selected to cover a range of functional tasks. Atzori et al. included 50 movements chosen from hand taxonomy, robotics, and rehabilitation literature to represent activities of daily living. These encompassed various hand postures, finger movements, wrist motions, and functional grasps. Jarque-Bou et al. similarly included 40 hand movements and grasps to capture common daily activities. Krasoulis et al. focused on a smaller set of six classes including different grasp types needed to manipulate objects. The researchers aimed to include movements relevant to prosthetic control and representative of real-world hand use.

While these datasets cover many functional hand movements, they do not include tasks specifically related to pouring liquids. The existing datasets focus on grasps, finger motions, and wrist movements, but lack the precise control and dynamic adjustments needed for manipulating containers and pouring accurately. This represents a gap in the current research, as pouring is an important activity of daily living that requires dexterous control. The present study aims to address this by developing a dataset and recognition methods focused on the hand movements and control strategies involved in pouring tasks. This will expand our understanding of myoelectric control for more complex, dynamic activities crucial for prosthesis users.

### 2.2. EMG-Based Hand Gesture Recognition

Thresholding is a fundamental method for detecting muscle activation, with single-threshold and double-threshold techniques being used. Double-threshold methods generally provide higher detection probability and allow more flexibility in balancing false alarms and detection rates. The signal-to-noise ratio should maximize the information content from the EMG signal while minimizing noise contamination.

Several classification methods have been applied to EMG signals for tasks such as gesture recognition and muscle fatigue analysis. K-nearest neighbor (kNN) is a non-parametric method that classifies data points based on the majority class of their k-nearest neighbors. Support vector machines (SVM) compute optimal classification functions to distinguish between classes. Linear discriminant analysis (LDA) creates hyperplanes to separate classes based on all data points. Naive Bayes classifiers use probability theory to compute the most probable class for new data points [14]. Artificial neural networks (ANN) have also been used for EMG classification, with the ability to learn complex relationships between inputs and outputs. Fuzzy logic systems have been applied to EMG analysis, offering advantages in handling contradictions in data and emulating human decision-making.

Deep learning methods have shown significant potential in improving sEMG classification for human–machine interaction applications [15]. Various deep neural network architectures have been employed, including convolutional neural networks (CNNs), recurrent neural networks (RNNs), autoencoders (AEs), and deep belief networks (DBNs). CNNs have demonstrated superior performance in hand gesture classification tasks, while RNNs, particularly LSTM and GRU variants, have been effective in processing temporal information within sEMG signals. Mixed network structures, combining different types of deep learning models, have shown improved performance by extracting both spatial and temporal features simultaneously. Deep learning approaches have also addressed challenges such as inter-subject and inter-session variability through transfer learning techniques. These methods offer advantages in automatic feature learning, multimodal sensor fusion, and robustness against disturbances like electrode shift and muscle fatigue. However, challenges remain in terms of computational cost and data dependency, which are active areas of research for future improvements in sEMG-based human–machine interfaces.

### 2.3. Recent Advances in Upper-Limb Motor Recovery and Replacement

#### 2.3.1. Hand-Wrist Rehabilitation

The development and application of surface electromyography (sEMG) sensors in rehabilitation technologies have seen significant innovations aimed at improving the accuracy and comfort of long-term muscle monitoring. This section reviews recent advancements in sEMG sensor technology, continuous motion prediction, and EMG-driven musculoskeletal models.

Flexible sEMG sensors employing graphene-based flexible electrodes on a polydimethylsiloxane (PDMS) substrate have demonstrated substantial improvements in hand-wrist rehabilitation [16]. These sensors integrate graphene transfer technology with a flexible printed circuit board for signal acquisition, offering enhanced flexibility and comfort. A recent study using an LSTM network reported a high accuracy of 98.81% in muscle strength prediction, proving its utility in controlling hand rehabilitation robots and assessing muscle strength.

The accuracy of sEMG signals in predicting continuous motion has been critically analyzed in a systematic review of both model-based and model-free approaches. The integration of muscle synergy and motor unit neural features with computational models such as CNN-LSTM and attention mechanisms has been highlighted [17]. The review also discusses the role of transfer learning in improving model generalizability across different subjects and sessions, despite existing challenges related to signal stability, environmental robustness, and the prediction of complex multi-joint movements.

Furthermore, an EMG-driven musculoskeletal model has been proposed to estimate wrist kinematics accurately, focusing on flexion/extension and radial/ulnar deviation [18]. This model, which utilizes mirrored bilateral movements, has shown high estimation accuracy in cases of unilateral transradial amputees and suggests potential for simultaneous and proportional control of multiple wrist kinematics.

These studies collectively advance our understanding of the potential and challenges of sEMG technology in rehabilitation settings. By enhancing sensor flexibility, accuracy in motion prediction, and the physiological relevance of musculoskeletal models, these technological advancements pave the way for more effective rehabilitation strategies. As these technologies continue to evolve, further research will be necessary to address the remaining challenges and fully exploit the capabilities of sEMG in clinical applications.

#### 2.3.2. Exoskeleton Control

Upper limb rehabilitation exoskeletons have emerged as promising devices to assist and restore motor functions in people with motor disabilities, as well as to augment human performance in able-bodied individuals. These exoskeletons can be broadly categorized into rigid exoskeletons with links and joints, and soft exosuits using flexible materials [19]. The mechanical design of exoskeletons has evolved from simple passive devices to more complex active systems with multiple degrees of freedom.

Upper limb exoskeletons have evolved significantly, integrating new actuation and control strategies to enhance rehabilitation. Traditional rigid exoskeletons often face limitations such as high weight and power consumption, restricting their usability outside clinical settings. To address this, EMG-driven exoneuromusculoskeletons have been developed, combining neuromuscular electrical stimulation (NMES) and soft pneumatic muscles to assist movement while promoting voluntary muscle activation [20].

Another approach focuses on postural synergy-based control, which leverages natural movement patterns to improve rehabilitation effectiveness. The Armule exoskeleton, for instance, simplifies control complexity by reducing degrees of freedom while ensuring movement assistance aligns with human biomechanics. Clinical studies have shown that synergy-based training can significantly enhance motor function in stroke patients [21].

While both approaches have shown promise, they target different rehabilitation needs: EMG-driven systems rely on user-initiated effort, whereas synergy-based designs provide structured movement guidance. Future work may benefit from hybrid models that integrate both strategies for adaptive rehabilitation.

#### 2.3.3. Stroke Rehabilitation

Stroke rehabilitation has seen significant advancements through the integration of electromyography (EMG) and electroencephalography (EEG) technologies. EMG-based systems have demonstrated promising results in decoding motor intentions from paretic muscles of stroke patients. These systems have been successfully integrated into rehabilitation robotics, leading to the development of innovative devices such as EMG-driven exoskeletons and functional electrical stimulation systems. EMG signals provide valuable insights into muscle function changes post-stroke, including reduced activation, altered motor unit firing rates, and changes in muscle fiber composition. Advanced signal processing techniques have been developed to quantify these alterations and improve the accuracy of motor intention detection [22]. While EMG-based approaches show great potential, EEG-based brain–computer interfaces (BCIs) offer an alternative, particularly for patients with severe paralysis or limited residual muscle activity. Recent research has explored hybrid brain–machine interfaces (hBMIs) that combine both EMG and EEG signals, showing improved performance in detecting motor intentions compared to single-modality approaches [19].

To further enhance the effectiveness of these systems, future efforts could focus on real-time adaptive algorithms that adjust to the patient’s progress during therapy sessions, and the development of lightweight, more ergonomic devices that can be comfortably worn for extended periods. Additionally, integrating machine learning models that predict recovery patterns could personalize treatment plans, optimizing rehabilitation outcomes. Despite these advancements, challenges remain in improving signal quality, developing more sophisticated algorithms, and addressing issues related to signal contamination and variability among patients.

## 3. The Proposed Approach

### 3.1. Sensory Data Acquisition System

**Figure 1:**
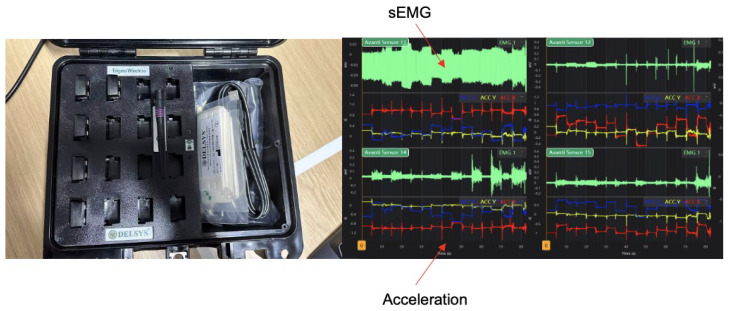
Delsys and Trigno EMG data collection system and multi-channel sample EMG waves. X: (mV) for sEMG and (G) for acceleration. Y: Time (s).

This research captured sEMG signals using the Delsys Trigno system (Delsys Inc., Boston, MA, USA), as shown in Figure 1. This system employs compact, wireless sensors equipped with four silver bar electrodes and a built-in amplifier. The reusable sensors are affixed directly to the forearm-wrist skin with double-sided adhesive tape, simplifying placement and eliminating the need for electrode gel or wet electrodes. The integrated electrodes minimize artifacts, which is crucial for dynamic activities like gait. Each sensor also includes a 3-axis accelerometer, enabling synchronized monitoring of EMG signals and inertial sensor data. Before further analysis, a customized synchronization system with routine filtering for physiological signals was used for data preprocessing. This advanced setup ensures high fidelity in signal capture, essential for accurate gesture recognition in rehabilitation applications. The ability to reliably detect nuanced muscle activations with minimal noise interference enhances the system’s utility in clinical settings, where precise motor control analysis is critical. Moreover, the portability and ease of use of the Trigno system allow for seamless integration into daily therapy sessions, promoting consistent usage and data collection over extended periods.

### 3.2. Experiment Protocol

The case study focuses on the strategic selection of muscles for sourcing EMG signals in gesture classification systems, particularly due to the complex musculature and tendons found in the forearm and wrist areas. These locations are selected as primary sites for EMG acquisition because they offer a detailed map of muscle activity essential for executing a broad spectrum of hand movements. This approach is advantageous for both able-bodied individuals and amputees, who may retain active residual muscles in their stumps capable of generating useful EMG signals. These signals are crucial for effectively controlling prosthetic limbs, thereby substantially enhancing the functionality and integration of assistive devices into the daily lives of users.

In this case study, EMG signals were collected from a 27-year-old male participant. The experiment adhering to these parameters received ethical approval from the University of Portsmouth Ethics Committee, ensuring compliance and safety in research practices under approval number TECH2024-CZ-01v5.

In tasks that require fine motor skills, such as pouring water or handling delicate materials, the detection of precise muscle activity is imperative. The forearm muscles and wrist tendons are rich sources of EMG data, providing a detailed spectrum of electrical signals that allows for the accurate identification of subtle gestures involved in such tasks. This capability is particularly crucial in the development of EMG-based human–computer interaction (HCI) technologies, which find applications in a variety of fields, ranging from rehabilitation to the creation of advanced assistive technologies and interactive gaming systems. This enhanced capability not only improves the functionality of devices but also aids in the seamless integration of technology into everyday life, promoting greater independence and quality of life for users.

Before data collection commenced, the participant was thoroughly briefed using an information sheet and a consent form to familiarize themselves with the steps involved in the collection process. To ensure consistency and reliability in the data collection, a brief demonstration was provided. The subject was seated in a neutral position, with the upper limb and elbow comfortably resting on the desk. They were provided with a detailed experimental protocol which they needed to understand and consent to before proceeding.

**Figure 2:**
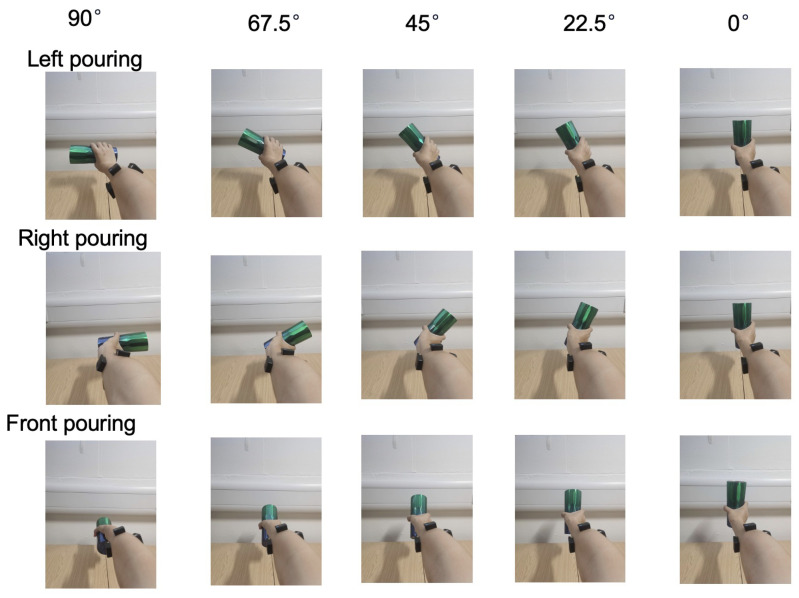
The scenario of three types of gestures for water pouring. Left/right/front pouring indicates pouring water to left/right/front.

The participant was then instructed to hold a water bottle and perform precise hand turns to the left, right, and front, demonstrating various angles—90°, 67.5°, 45°, and 22.5°—as outlined in Figure 2. Each position was maintained for 5 s, followed by a 5 s rest period to minimize muscle fatigue and ensure consistent data quality.

The Trigno wireless system, equipped with four channels located at the front, back, left, and right of the forearm, was used to capture EMG data. To maximize the accuracy and reproducibility of the data, four sensors were strategically positioned along the thickest part of the forearm. Sensor 1 was centrally placed when the palm faced upwards, with the remaining sensors—sensors 2, 3, and 4—arranged sequentially in a clockwise direction. This setup allowed for comprehensive coverage of muscle activity across different segments of the forearm, enhancing the sensitivity and specificity of the EMG readings.

Before attaching the Delsys sensors with double-sided tape, the participant’s arm was carefully cleaned and allowed to rest and relax for at least 30 s. This preparation was crucial to ensure that the skin surface was optimal for sensor adhesion and signal clarity. Throughout the experiment, each muscle activation was followed by a 5 s break, allowing the muscles to fully relax before the next data capture to prevent any signal overlap or muscle strain.

Each session began with a careful check of the sEMG sensors to confirm that they were operational and free from electrical noise, guaranteeing the reliability of the recordings. After the data collection phase, the acquired sEMG signals were meticulously analyzed to verify the correctness of each channel and the integrity of the signals, ensuring that the data were precise and suitable for further analysis. This rigorous process aided in obtaining high-quality EMG data, which are crucial for the successful application and development of gesture recognition systems.

### 3.3. Signal Processing

The EMG signals are initially captured via electrodes affixed to the forearm-wrist skin surface. To refine these raw EMG signals for subsequent analysis, both a powerline filter and a Butterworth filter are employed, facilitating the generation of a processed EMG signal stream dedicated to hand motion recognition. The analysis adopts an overlapping windowing scheme characterized by a window length of 250 ms and an overlap of 50 ms. This configuration is strategically chosen to create a dense decision stream, optimizing the temporal resolution necessary for effective post-processing.The rationale behind selecting a 250 ms window length primarily revolves around the trade-off between capturing sufficient muscle activity data and maintaining manageable computational complexity. This duration is long enough to encompass the full cycle of most hand gestures while short enough to prevent the inclusion of irrelevant signal variations, thereby ensuring the precision of gesture detection.

The 200 ms overlap—constituting 80% of the window length—is meticulously calculated to enhance the continuity and frequency of data updates. This overlap is critical for capturing the dynamism in hand motions, allowing for smoother transitions in gesture recognition and reducing the likelihood of missing brief muscle activations. It significantly improves the system’s responsiveness to rapid movements, a vital feature for real-time applications. Moreover, the system integrates both handcrafted features and deep learning models to process each EMG segment. Handcrafted features are extracted to provide a foundational understanding of the signal characteristics, which include, but are not limited to, statistical metrics such as mean absolute values and waveform lengths. Concurrently, these features are concatenated with inputs derived from deep learning models to enrich the input stream fed into the model. This hybrid approach leverages the strengths of traditional signal processing techniques and advanced machine learning models, ensuring robust and accurate hand motion classification [23].

In sEMG-based applications, time-domain and autoregressive features have played a dominant role for decades for their robustness in performance across subjects and scenarios [24]. The handcrafted feature set in this research includes time-domain features such as root mean square, variance, waveform length, and zero crossings. These features were selected for their simple extraction process and robust performance, providing an effective balance with time consumption. This balance is crucial for minimizing any perceivable delay for users. Specifically, with the *i*-th sample in the EMG stream denoted as $x_{i}$, the root mean square is a feature to evaluate EMG signal amplitude, as shown in Equation (1).

(1) $$RMS=\sqrt{\frac{1}{N}\sum_{i=1}^{N}x_{i}^{2}}$$

Variance, similar to features that include simple square integral, mean power, and total power, is calculated using Equation (2).

(2) $$VAR=\frac{1}{N-1}\sum_{i=1}^{N}x_{i}^{2}$$

Zero crossings, which refer to the number of times a signal crosses the zero axis and are somewhat related to the frequency characteristics of EMG signals, are defined in Equation (3).

(3) $$ZC=\sum_{i=1}^{N}\mathrm{sgn}\left(-x_{i}x_{i+1}\right)$$

where the sign function sgn is defined as follows:

(4) $$\mathrm{sgn}\left(x\right)=\left\{\begin{array}{cc} 1 & \mathrm{if}\;x>\varepsilon \\ 0 & \mathrm{if}\;x\leq \varepsilon \end{array}\right.$$

The waveform length, which is the cumulative waveform of the EMG signal’s length, is calculated as described in Equation (5).

(5) $$WL=\sum_{i=1}^{N-1}\left|x_{i+1}-x_{i}\right|$$

### 3.4. Convmixer

**Figure 3:**
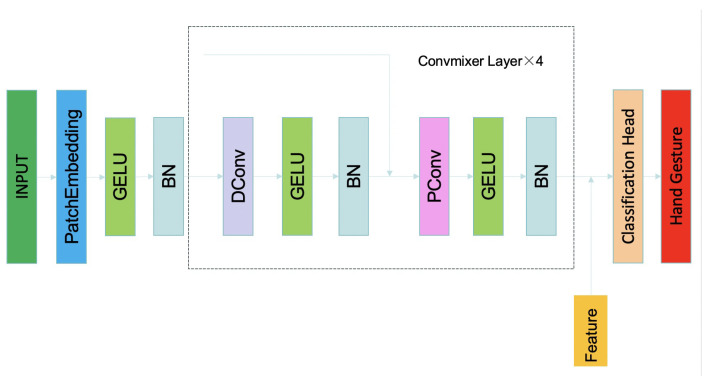
The deep structure of hand gestures. The input data consists of 13 labels, with each label containing 100 × 3 sets of data (No. 5 and No. 10 are both ‘hold’, so they both count as No. 5, so No. 5 will have 100 × 3 × 2). Each dataset has four channels, with 481 sEMG data points per window. (481 point is cause by the frequency of the data collect, 1925.9259 Hz × 250 ms for each window) DConv: depthwise convolution. BN: batch normalization.

ConvMixer is a simple yet effective architecture that independently mixes the spatial and channel locations of patch embeddings using standard convolution operations. One of the main advantages of this model is its simplicity while still outperforming traditional vision models such as ResNet and some variants of Vision Transformer and MLP-Mixer with similar parameter counts and dataset sizes. Additionally, ConvMixer significantly enhances performance by using large kernel sizes for convolutions, inspired by the large receptive fields of Vision Transformers and MLP-Mixers, demonstrating that large-kernel convolutions are highly effective in mixing spatial information in visual tasks [25]. Figure 3 shows the deep structure of the ConvMixer model applied to hand gesture classification. The diagram illustrates the flow of data from the input stage through multiple ConvMixer layers, incorporating depthwise and pointwise convolutions, GELU activations, and batch normalization. The final output is fed into the classification head, producing the hand gesture predictions.

In the context of noisy far-field keyword spotting, ConvMixer is highly effective. The model employs a novel convolutional network encoder with a mixer module that offers a strong alternative to attention mechanisms. The mixer unit computes the weighted feature interaction of the global channel, allowing the flow of information with varying importance. This architecture is particularly efficient for memory and computation, making it suitable for small devices. Curriculum-based multi-condition training enhances the model’s noise robustness, achieving state-of-the-art accuracy among small models when tested on the Google Speech Commands V2 dataset [26].

ConvMixer operates through several key components: patch embedding, depthwise convolution, pointwise convolution, and batch normalization. The input image is initially divided into patches, which are then linearly embedded as shown in Equation (6). The ConvMixer block itself consists of depthwise convolution, which is a grouped convolution with groups equal to the number of channels, followed by pointwise convolution, shown in Equations (7) and (8), respectively. Each convolution is followed by an activation function and batch normalization, described in Equation (9). This combination allows ConvMixer to effectively mix spatial and channel information while maintaining computational efficiency. The use of large kernel sizes in depthwise convolutions helps in mixing distant spatial information, similar to the large receptive fields in Vision Transformers and MLP-Mixers.

**Patch Embedding**

(6) $$Y=\mathrm{Conv}2D(X,W_{\mathrm{patch}},\mathrm{stride}=p)$$

**Depthwise Convolution**

(7) $$Y=\mathrm{Conv}2D(X,W_{\mathrm{depthwise}},\mathrm{groups}=C,\mathrm{padding}=\mathrm{same})$$

**Pointwise Convolution**

(8) $$Y=\mathrm{Conv}2D(X,W_{\mathrm{pointwise}},\mathrm{kernel}\_\mathrm{size}=1)$$

**Batch Normalisation**

(9) $$Y=\frac{X-\mu }{\sqrt{\sigma^{2}+\epsilon }}\cdot \gamma +\beta$$

Given its robust performance in handling noisy conditions and its efficient architecture, ConvMixer can be effectively applied to sEMG action recognition classification tasks, where similar challenges of noise and computational efficiency are prevalent.

### 3.5. Ganular Computing

Granular computing (GrC) is a paradigm of information processing that takes advantage of the concept of granules, which are clusters of similar or related information entities. These granules can be subsets, classes, objects, clusters, or elements of a universe, and they are formed based on distinguishability, similarity, or functionality [27]. GrC focuses on structured thinking and problem-solving by organizing information at multiple levels of granularity, helping to simplify complex problems, improve clarity, and manage uncertainty. The principles of GrC are applied in various fields, including artificial intelligence, machine learning, and data compression, to improve the efficiency and effectiveness of information processing.

In practice, GrC is applied across various domains such as artificial intelligence, machine learning, data compression, and even more broadly in system theory and cognitive science. By structuring data and concepts into layers or hierarchies of granularity, GrC enables more efficient computation and better decision-making. For instance, in machine learning, it aids in creating more manageable datasets by grouping large sets of data into interpretable clusters, which can then be analyzed or processed separately. In artificial intelligence, granular computing helps to model human-like reasoning and decision-making processes by approximating the way people process information in layers—from broad to specific. This tiered approach facilitates the handling of complex and ambiguous information, making GrC a vital tool in the advancement of computational intelligence and information sciences.

In the context of sEMG-based action recognition, the ConvMixer model can be combined with granular computing to achieve more precise differentiation of actions. ConvMixer, a convolutional architecture that operates in patches and separates the mixing of spatial and channel dimensions, is effective in various tasks due to its simplicity and performance [25]. By integrating GrC, we can leverage the hierarchical structure of granules to manage the inherent correlations among different actions in sEMG data. This approach allows for the construction of granules that capture the essential features of each action at different levels of granularity, thereby improving the model’s ability to distinguish between similar actions. The use of GrC in this manner can enhance the accuracy of action recognition by focusing on the most relevant aspects of the data and reducing the impact of noise and irrelevant details. This method is particularly useful in datasets where many actions exhibit significant correlations, as it helps to isolate and identify the unique characteristics of each action more effectively.

**Figure 4:**
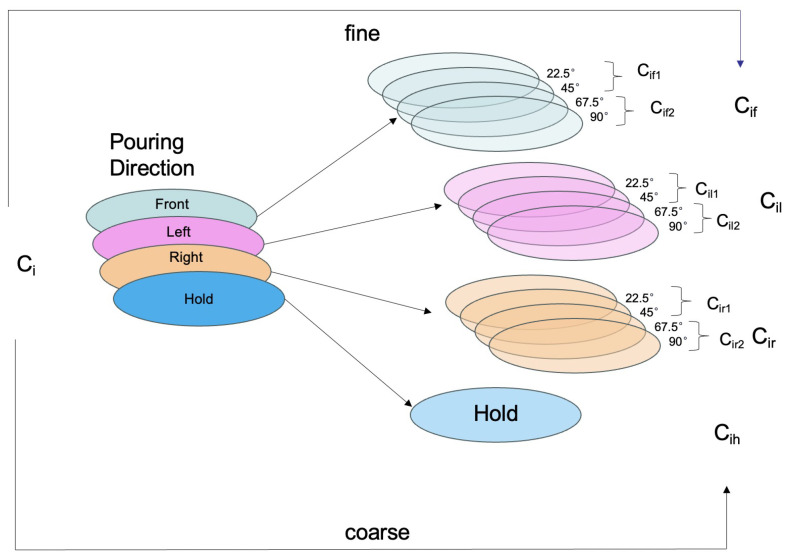
Transferring a pouring direction to a granulated angle via information two granulations.

In gesture recognition, granular computing is employed to refine the classification of complex gestures. As illustrated in Figure 4, the training dataset consists of various samples categorized into four primary gesture types: front, left, and right pouring directions, and holding. To enhance the classifier’s accuracy, each primary gesture category $C_{j}$ is subdivided into four granules based on the angle of execution. For instance, the action of pouring forward is segmented into subclasses at 22.5°, 45°, 67.5°, and 90°. This granulation generates new subclasses $C_{if}$, $C_{il}$, and $C_{ir}$ for each initial category, improving the classifier’s ability to detect subtle variations within a gesture.

This granularity not only enhances classification accuracy but also allows the classifier to identify finer nuances in gesture dynamics. Furthermore, a coarsening function is defined to map these granulated subclasses $C_{ij}$ back to their original primary categories $C_{i}$. For example, all angle-specific subclasses of forward pouring can be coarsened back into a single “Front” category. This approach accommodates both detailed analysis of specific gesture components and the flexibility to adjust the level of detail for more simplified outputs, facilitating efficient and adaptable processing in complex gesture recognition tasks [28].

While the above describes a four-subclass granulation for each gesture category ($C_{if},C_{il},C_{ir}$ subdivided by 22.5°, 45°, 67.5°, and 90°), we also introduce a simplified **two-class granulation**—referred to as *granular 2*. In this scheme, each primary gesture category is split into two angle-based groups, yielding subclasses like $C_{if1},C_{if2},C_{il1},C_{il2},C_{ir1},C_{ir2}$. By merging angles (e.g., grouping 22.5° and 45° as one subset, and 67.5° and 90° as another), the model can operate on a coarser level of detail while still retaining some granularity. This two-level approach can be beneficial in scenarios that demand fewer gesture classes or simpler angle distinctions, offering flexibility in balancing classification detail and computational complexity.

### 3.6. Implementation

This implementation emphasizes the integration of hand-crafted feature extraction into the training of a ConvMixer model, enhancing its capability to analyze and interpret signal data more effectively. The FeatureExtractor component is crucial as it is specifically designed to compute various signal features such as root mean square, variance, waveform length, and zero-crossing rates. These features are instrumental in capturing essential characteristics of the input data, which can significantly influence the model’s learning and generalisation performance. By incorporating these hand-crafted features, the model not only relies on raw data but also uses these engineered attributes to improve its predictive accuracy. This dual approach leverages both deep learning techniques and traditional signal processing methods, ensuring a more robust model capable of handling complex patterns in the data.

During the training process, these extracted features are fed into PatchEmbeddings, which further processes them through 2D convolution layers, followed by GELU activation and batch normalisation. This helps in embedding the features into a format suitable for the sequential layers of the ConvMixer, thus enhancing the overall data representation before it undergoes further analysis in the depthwise and pointwise convolutional layers of the ConvMixerLayer. The integration of these handcrafted features is particularly useful in applications where signal integrity and detailed feature analysis is crucial, such as in audio processing, biomedical signal analysis, or any scenario where precise feature extraction can lead to better insights and outcomes. The entire framework is designed to be flexible, allowing for the easy addition or modification of feature extraction methods based on specific project needs or data characteristics.

### 3.7. MBTCN and CAPSULE

MBTCN is an advanced method known for leveraging a “local extraction and global integration” strategy to capture both cross-correlation and temporal-correlation in data. By dividing variables into sub-blocks based on process mechanisms, it employs 1D-CNN to extract temporal features within each sub-block, ensuring closely related variables are processed together. This design overcomes the limitations of traditional 2D-CNNs and enhances the model’s ability to learn fine-grained features. Its ability to analyze multivariate time-series data makes it highly applicable to sEMG processing, where capturing temporal dependencies and correlations between multiple muscle signals is critical. MBTCN has demonstrated superior fault diagnosis performance, achieving a 0.962 accuracy on the TE process [29].

Capsule networks, introduced by Hinton et al., represent a notable advancement in neural architectures, addressing limitations of CNNs such as the inability to model spatial hierarchies and lack of rotational invariance [30]. Capsules group neurons into vectors that encode properties and spatial relationships of features, offering improved representational capabilities. Features like squashing and dynamic routing enhance invariance more effectively than max-pooling in CNNs. Originally developed for image processing tasks, such as achieving a 0.25% test error on the MNIST dataset without data augmentation, capsule networks are also applicable to sEMG data. The spatial hierarchies and temporal dependencies in sEMG signals resemble those in image data, enabling capsule networks to effectively model complex feature relationships for gesture recognition and similar tasks.

These two models were selected as baselines to provide robust comparisons for validating the proposed methods. MBTCN’s ability to handle multivariate time-series data and effectively capture temporal and cross-variable correlations makes it a strong benchmark for sEMG processing tasks. Similarly, capsule networks, with their capacity to model spatial hierarchies and feature relationships, offer a complementary perspective for evaluating performance. By leveraging these established models, the proposed methods can be rigorously assessed against well-recognized approaches, ensuring a comprehensive validation of their effectiveness and efficiency in sEMG-based applications. The integration of these models provides a solid foundation for assessing the nuances of gesture recognition, enhancing the empirical robustness of the research and ensuring that advancements in sEMG signal processing continue to push the boundaries of what is possible in rehabilitation technologies and beyond.

### 3.8. Feature Fusion

In the field of deep learning, particularly in image processing, the importance of feature fusion has been well-established. Combining hand-crafted features with neural network-generated features can significantly enhance classification performance by providing complementary information that networks alone may overlook. Hand-crafted features such as local binary patterns (LBP), histogram of oriented gradients (HOG), and scale-invariant feature transform (SIFT) capture specific aspects like texture, shape, and fine details. These features are particularly useful in addressing limitations of neural networks, such as handling fine-grained patterns or limited training data [31]. For example, Jochumsen demonstrated that combining CNN features with hand-crafted ones (e.g., colour histograms, HOG, LBP, and dense-SIFT) improved classification accuracy on the Cifar10 dataset. While HOG alone achieved 52.71% accuracy, and a combination of all hand-crafted features reached 61.18%, integrating these with CNN features raised accuracy from 74.83% to 76.51% [32]. This highlights the complementary role of hand-crafted features in addressing neural network limitations. Further, proposed a fusion system combining deep CNN features with hand-crafted ones, achieving state-of-the-art results across various datasets. The study emphasized that each feature type captures unique aspects of data, and their combination significantly outperformed standard methods (*p* = 0.01) [31].

This proven strategy of feature fusion in image processing can be effectively adapted to sEMG-based gesture recognition. Recent research has highlighted the feasibility of leveraging feature fusion to enhance performance in sEMG-related recognition tasks [33,34]. By integrating hand-crafted features with neural networks, nuanced signal patterns in sEMG data can be more accurately classified, enabling robust performance even in complex and challenging scenarios.

### 3.9. Accuracy, Sensitivity, and Specificity

In this study, we investigate the classification of sEMG signals associated with pouring actions. To rigorously assess the performance of the proposed models, we employ three widely recognized evaluation metrics: accuracy, sensitivity, and specificity. These metrics provide not only an overall measure of the model’s performance but also a detailed insight into its ability to correctly classify positive instances and effectively reject negative ones.

Accuracy is defined as the ratio of correctly classified instances (both positive and negative) to the total number of instances:

(10) $$Accuracy=\frac{TP+TN}{TP+TN+FP+FN},$$

where TP (true positives) denotes the number of correctly identified positive cases, TN (true negatives) represents the number of correctly identified negative cases, FP (false positives) indicates the number of negative cases incorrectly classified as positive, and FN (false negatives) is the number of positive cases incorrectly classified as negative.

Sensitivity, also referred to as recall, quantifies the model’s ability to correctly identify positive instances:

(11) $$Sensitivity=\frac{TP}{TP+FN}.$$

Specificity evaluates the model’s capacity to correctly classify negative instances:

(12) $$Specificity=\frac{TN}{TN+FP}.$$

These metrics are critical for our analysis as they provide a comprehensive evaluation of the model’s discriminative ability. Notably, our experimental results demonstrate that the ConvMixer model under the Granular1 configuration achieves superior performance across all three metrics, thereby underscoring its efficacy in capturing fine-grained features essential for precise signal analysis and classification.

## 4. Results

The data from Table 1 illustrate that the integration of granular features leads to performance improvements in all three models. Specifically, the MBTCN model’s accuracy increased from 0.9762 to 0.9857, the CAPSULE model from 0.1889 to 0.4794, and the ConvMixer model from 0.9821 to 0.9929 with the incorporation of granular features (FG-1). In particular, the ConvMixer model exhibits a superior enhancement, consistently achieving the highest performance across different granular settings, which underscores its effectiveness in leveraging these additional features for enhanced task performance.

The data from Table 2 further demonstrate that the integration of granular features (FG) generally yields better performance compared to using only standard features (F) across various models. For instance, the SVM model shows an increase from 0.8775 to 0.9704, the random forest from 0.7668 to 0.9213, and the LDA from 0.7391 to 0.9387. Similarly, the MBTCN model improved from 0.8030 to 0.9555 and the CAPSULE model from 0.8830 to 0.9378. Notably, the ConvMixer model’s ability to categorize distinct classes is markedly enhanced, with its accuracy increasing from 98.21% to 99.29% when granular features are applied. This trend not only confirms the substantial impact of finer-grained features on boosting predictive accuracy but also highlights the superior capability of the ConvMixer model in effectively exploiting these features for sEMG signal analysis.

In addition, it is important to understand the evaluation metrics used in these experiments. Accuracy represents the overall proportion of correctly classified samples among all samples, reflecting the general performance of the model. Sensitivity, also known as recall, indicates the model’s ability to correctly identify positive samples, while specificity measures the capability of correctly excluding negative samples. Based on the data presented, the ConvMixer model under the Granular1 (FG-1) setting achieves the best performance in terms of accuracy, sensitivity, and specificity. This further demonstrates its superior effectiveness in utilizing fine-grained features for accurate signal analysis and classification.

The findings of this research highlight several key insights into the application of deep learning models to rehabilitative gesture recognition. Firstly, the integration of granular computing into the ConvMixer architecture enables a nuanced analysis of sEMG signals at various levels of granularity. This capability is critical in clinical settings where distinct phases of a gesture, such as those involved in pouring, must be accurately recognized and differentiated. Granular computing achieves this by segmenting signal processing into manageable parts, thereby enhancing the model’s ability to focus on significant signal variations that directly impact gesture recognition accuracy.

In addition, the use of handcrafted features—derived from expert knowledge of muscle function and biomechanics—significantly augments the learning process by providing the model with predefined insights essential for interpreting complex muscle activities. Although this reliance on hand-crafted features may limit flexibility in recognizing new or uncharacterized gestures without further re-engineering, it substantially improves both the efficiency and accuracy of the recognition process.

**Figure 5:**
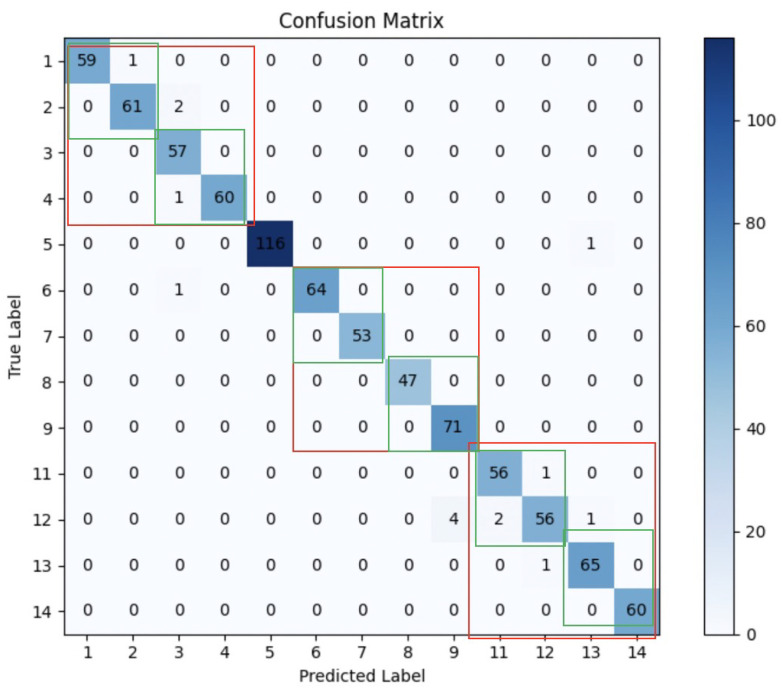
Confusion matrix granular computing. X: predicted labels. Y: true labels (1–4 left pouring, 6–9 right pouring, 11–14 front pouring, 5 hold). RED Square: granular 1. GREEN Square: granular 2.

The confusion matrices provided in Figure 5 further highlight the improvements in model performance when granular features are incorporated. Notably, the ConvMixer model’s enhanced ability to categorize distinct classes, with accuracy rising from 98.21% to 99.29%, demonstrates its superior performance. Despite occasional misclassifications, most errors occur within adjacent categories, which is particularly advantageous in applications where high precision is paramount, such as prosthetic hand manipulation.

Moreover, the improvement in ConvMixer’s performance underscores the critical role of granular computing in capturing subtle differences within the data to boost predictive accuracy. The predominance of misclassifications within closely related categories minimizes the potential for significant operational errors, ensuring safer and more reliable application in scenarios where error consequences can be severe.

## 5. Conclusions

In this study, we have developed and evaluated a specialized sEMG-based gesture recognition system designed to enhance the functionality of rehabilitation technologies, specifically for pouring tasks. Our approach integrates sophisticated computational strategies, including hand-crafted features and granular computing, into the ConvMixer architecture, which has traditionally excelled in general image recognition tasks. By adapting this architecture to the specific dynamics of muscle-generated electrical signals, our system demonstrates marked improvements over conventional models.

The empirical evidence from our experiments shows that our enhanced ConvMixer model outperforms established models such as MBTCN and CAPSULE, as well as standard machine learning algorithms such as SVM and random forest. Moreover, the incorporation of additional evaluation metrics—accuracy, sensitivity, and specificity—provides a more comprehensive assessment of the model’s performance, further substantiating the superior efficacy of the ConvMixer with granular computing in precise gesture recognition.

In this study, we are pioneers in utilizing a database tailored specifically for tasks like pouring, a common yet complex rehabilitation activity. As there is no established state of the art (SOTA) for direct comparison within this specific dataset, our approach establishes a unique benchmark in the field. This distinction underscores the innovative nature of our approach and the novel application of the ConvMixer architecture in this context.

## 6. Future Work

Future research could explore automating feature extraction in sEMG-based gesture recognition to reduce reliance on expert-defined features, potentially enhancing generalisability. Deep learning and self-learning methods might enable models to extract features directly from raw sEMG signals, improving accuracy and efficiency across diverse movements. Additionally, optimizing computational frameworks to address the demands of granular computing may support scalability and real-time performance, particularly in precision-critical rehabilitation settings. Granular computing could also enhance sEMG classification through multi-scale analysis, improving accuracy while accelerating training and inference. Its ability to decompose problems into granularities may enable parallel processing, benefiting tasks like motion control, gesture tracking, and posture recognition.

Further refining the granularity of feature extraction could involve analyzing continuous variations in muscle activation angles or increasing the resolution of temporal and spatial muscle activity patterns. This enhanced granularity could allow for a more detailed understanding of nuanced muscle movements, offering deeper insights into complex gesture dynamics. By integrating granular computing with advanced machine learning techniques, there is potential to develop robust, adaptive systems for applications ranging from assistive device control to personalized healthcare solutions, enabling more precise and dynamic response capabilities in real-time applications.

Given the absence of a direct state-of-the-art comparison within this specialized dataset, future research is encouraged to utilize similar databases to establish robust benchmarks against our findings. We invite further studies to compare and possibly enhance the models based on these specialized datasets. Such collaborative and comparative efforts would validate and potentially refine the computational techniques used in sEMG-based gesture recognition, leading to advancements in academic research and practical applications.

## Figures and Tables

**Table 1:** Performance metrics on original data with feature and feature granular settings. **OD**: original data. **F**: feature. **FG**: feature granular.

Model	OD with F (Original)			OD with FG-1 (Granular1)			OD with FG-2 (Granular2)		
	Accuracy	Sensitivity	Specificity	Accuracy	Sensitivity	Specificity	Accuracy	Sensitivity	Specificity
MBTCN	0.9762	0.9752	0.9980	0.9857	0.9861	0.9954	0.9794	0.9794	0.9966
CAPSULE	0.1889	0.1891	0.9318	0.4794	0.4440	0.8259	0.2873	0.2903	0.8813
**CONVMIXER**	**0.9821**	**0.9821**	**0.9985**	**0.9929**	**0.9927**	**0.9975**	**0.9881**	**0.9882**	**0.9980**

**Table 2:** Results for feature and original data. **F**: feature. **FG**: feature granular. **OD**: original data. **ODG**: original data granular.

Model	F	FG	OD	ODG
SVM	0.8775	0.9704	-	-
Random Forest	0.7668	0.9213	-	-
LDA	0.7391	0.9387	-	-
MBTCN	0.8030	0.9555	0.3683	0.5921
CAPSULE	0.8830	0.9378	0.2167	0.4955
**CONVMIXER**	0.6889	0.8281	**0.9512**	**0.9798**

## Data Availability

The personal data supporting the findings of this study are stored on the University of Portsmouth’s cloud server. Access to these data is restricted and available from the corresponding author upon reasonable request.

## References

[1] Trigili E., Grazi L., Crea S., Accogli A., Carpaneto J., Micera S., Vitiello N., Panarese A. (2019). Detection of movement onset using EMG signals for upper-limb exoskeletons in reaching tasks. J. Neuroeng. Rehabil..

[2] Hudgins B., Parker P., Member S., Member S. (1993). A New Strategy for Multifunction Myoelectric Control. IEEE Trans. Biomed. Eng..

[3] Ren Z., Meng J., Yuan J. Depth camera based hand gesture recognition and its applications in human-computer-interaction. Proceedings of the 2011 8th International Conference on Information, Communications & Signal Processing.

[4] Kiguchi K., Tanaka T., Fukuda T. (2004). Neuro-fuzzy control of a robotic exoskeleton with EMG signals. IEEE Trans. Fuzzy Syst..

[5] Çari Kiliboz N., Güdükbay U. (2015). A hand gesture recognition technique for human-computer interaction. J. Vis. Commun. Image Represent..

[6] Tsai T.H., Huang C.C., Zhang K.L. (2020). Design of hand gesture recognition system for human-computer interaction. Multimed. Tools Appl..

[7] Qin J., Martínez L., Pedrycz W., Ma X., Liang Y. (2023). An overview of granular computing in decision-making: Extensions, applications, and challenges. Inf. Fusion.

[8] Zhang H., Zhou Q.Q., Chen H., Hu X.Q., Li W.G., Bai Y., Han J.X., Wang Y., Liang Z.H., Chen D. (2023). The applied principles of EEG analysis methods in neuroscience and clinical neurology. Mil. Med. Res..

[9] Ahmed S., Kallu K.D., Ahmed S., Cho S.H. (2021). Hand gestures recognition using radar sensors for human-computer-interaction: A review. Remote Sens..

[10] Atzori M., Gijsberts A., Castellini C., Caputo B., Hager A.G.M., Elsig S., Giatsidis G., Bassetto F., Müller H. (2014). Electromyography data for non-invasive naturally-controlled robotic hand prostheses. Sci. Data.

[11] Krasoulis A., Kyranou I., Erden M.S., Nazarpour K., Vijayakumar S. (2017). Improved prosthetic hand control with concurrent use of myoelectric and inertial measurements. J. Neuroeng. Rehabil..

[12] Jarque-Bou N.J., Atzori M., Müller H. (2020). A large calibrated database of hand movements and grasps kinematics. Sci. Data.

[13] Krasoulis A., Vijayakumar S., Nazarpour K. (2019). Effect of User Practice on Prosthetic Finger Control with an Intuitive Myoelectric Decoder. Front. Neurosci..

[14] Venugopal G., Navaneethakrishna M., Ramakrishnan S. (2014). Extraction and analysis of multiple time window features associated with muscle fatigue conditions using sEMG signals. Expert Syst. Appl..

[15] Xiong D., Zhang D., Zhao X., Zhao Y. (2021). Deep Learning for EMG-based Human-Machine Interaction: A Review. IEEE/CAA J. Autom. Sin..

[16] Liu C., Li J., Zhang S., Yang H., Guo K. (2022). Study on Flexible sEMG Acquisition System and Its Application in Muscle Strength Evaluation and Hand Rehabilitation. Micromachines.

[17] Wei Z., Zhang Z.Q., Xie S.Q. (2024). Continuous Motion Intention Prediction Using sEMG for Upper-Limb Rehabilitation: A Systematic Review of Model-Based and Model-Free Approaches. IEEE Trans. Neural Syst. Rehabil. Eng..

[18] Zhao Y., Li Z., Zhang Z., Qian K., Xie S. (2023). An EMG-driven musculoskeletal model for estimation of wrist kinematics using mirrored bilateral movement. Biomed. Signal Process. Control.

[19] Xu P., Xia D., Li J., Zhou J., Xie L. (2022). Execution and perception of upper limb exoskeleton for stroke patients: A systematic review. Intell. Serv. Robot..

[20] Nam C., Rong W., Li W., Cheung C., Ngai W., Cheung T., Pang M., Li L., Hu J., Wai H. (2022). An Exoneuromusculoskeleton for Self-Help Upper Limb Rehabilitation after Stroke. Soft Robot..

[21] He C., Xiong C.H., Chen Z.J., Fan W., Huang X.L., Fu C. (2021). Preliminary Assessment of a Postural Synergy-Based Exoskeleton for Post-Stroke Upper Limb Rehabilitation. IEEE Trans. Neural Syst. Rehabil. Eng..

[22] Yang H., Wan J., Jin Y., Yu X., Fang Y. (2022). EEG- and EMG-Driven Poststroke Rehabilitation: A Review. IEEE Sens. J..

[23] la Fuente C.D., Martinez-Valdes E., Priego-Quesada J.I., Weinstein A., Valencia O., Kunzler M.R., Alvarez-Ruf J., Carpes F.P. (2021). Understanding the effect of window length and overlap for assessing sEMG in dynamic fatiguing contractions: A non-linear dimensionality reduction and clustering. J. Biomech..

[24] Phinyomark A., Quaine F., Charbonnier S., Serviere C., Tarpin-Bernard F., Laurillau Y. (2013). EMG feature evaluation for improving myoelectric pattern recognition robustness. Expert Syst. Appl..

[25] Trockman A., Kolter J.Z. (2022). Patches are all you need?. arXiv.

[26] Ng D., Chen Y., Tian B., Fu Q., Chng E.S. (2022). Convmixer: Feature interactive convolution with curriculum learning for small footprint and noisy far-field keyword spotting. Proceedings of the ICASSP, IEEE International Conference on Acoustics, Speech and Signal Processing—Proceedings.

[27] Yao J.T., Vasilakos A.V., Pedrycz W. (2013). Granular computing: Perspectives and challenges. IEEE Trans. Cybern..

[28] Fang Y., Zhou D., Li K., Ju Z., Liu H. (2021). Attribute-Driven Granular Model for EMG-Based Pinch and Fingertip Force Grand Recognition. IEEE Trans. Cybern..

[29] He Y., Shi H., Tan S., Song B., Zhu J. (2021). Multiblock temporal convolution network-based temporal-correlated feature learning for fault diagnosis of multivariate processes. J. Taiwan Inst. Chem. Eng..

[30] Xi E., Bing S., Jin Y. (2017). Capsule Network Performance on Complex Data. arXiv.

[31] Liu C.j., Marie D., Fredrick A., Bertram J., Utley K., Fess E.E. (2017). Predicting hand function in older adults: Evaluations of grip strength, arm curl strength, and manual dexterity. Aging Clin. Exp. Res..

[32] Jochumsen M., Waris A., Kamavuako E.N. (2018). The effect of arm position on classification of hand gestures with intramuscular EMG. Biomed. Signal Process. Control.

[33] Khushaba R.N., Phinyomark A., Al-Timemy A.H., Scheme E. (2020). Recursive Multi-Signal Temporal Fusions with Attention Mechanism Improves EMG Feature Extraction. IEEE Trans. Artif. Intell..

[34] Zhao L., Niu X., Wang L., Niu J., Zhu X., Dai Z. (2023). Stress Detection via Multimodal Multitemporal-Scale Fusion: A Hybrid of Deep Learning and Handcrafted Feature Approach. IEEE Sens. J..

